# The Cutaneous Leishmaniasis and the Sand Fly: Knowledge and Beliefs of the Population in Central Morocco (El Hajeb)

**DOI:** 10.1155/2020/1896210

**Published:** 2020-11-18

**Authors:** Karima El-Mouhdi, Abdelkader Chahlaoui, Mohammed Fekhaoui

**Affiliations:** ^1^Geobiodiversity and Natural Patrimony (GEOPAC), Scientific Institute, Mohammed V University, Rabat, Morocco; ^2^Natural Resources Management and Development Team, Laboratory of Health and Environment, Faculty of Sciences, Moulay Ismail University, Meknes, Morocco

## Abstract

**Background:**

Cutaneous leishmaniasis is a neglected parasitic dermal disease transmitted to humans through the bite of an infected female sand fly. Morocco hopes to eliminate all forms of leishmaniasis by 2030. These dermatoses pose a real public health problem in the country. Although the information is available on the disease, individual knowledge of cutaneous leishmaniasis and sand fly is not yet developed. Exploring people's beliefs and popular behaviours about cutaneous leishmaniasis and its vector allows health officials to know the sociocultural aspects of the disease and to improve prevention and control actions.

**Objectives:**

To identify the knowledge of cutaneous leishmaniasis and its vector in the population in central Morocco.

**Methods:**

Based on the epidemiological data of leishmaniases in the province of El Hajeb, we conducted a field survey and personal interviews in April and May 2019, among 281 persons belonging to the localities where leishmaniases were registered.

**Results:**

Our results show that the participants use the concept of “Chniwla” (61.6%) for sand fly and the concept of “Hboub Chniwla” (50.8%) for cutaneous leishmaniasis; 24.6% of the respondents do not know how the disease is transmitted to humans and 43.7% use traditional treatments and home remedies to cure themselves. 44% of participants believe that sand fly does not transmit the disease to humans and only 6.4% were aware of their responsibility in vector control.

**Conclusions:**

The study concluded that there is a need to simplify the scientific terminology in the health education of citizens regarding these dermatoses and their vector by integrating the popular concepts obtained in this study to raise public awareness and facilitate their involvement as active actors in the prevention of cutaneous leishmaniasis.

## 1. Introduction

Cutaneous leishmaniasis (CL) is a neglected dermal disease caused by *Leishmania* parasites transmitted to humans through the bite of an infected female sand fly [1]. Morocco is considered by the World Health Organization (WHO) as a country with a high burden of CL with an incidence rate of 5.62% and a population at risk of 14% [2]. Epidemiological data from the Moroccan Ministry of Health show that the vast majority of leishmaniasis cases are of the cutaneous type (97%) while the rest are of the visceral type (3%) [3]. The number of new cases of leishmaniasis registered at the national level has increased with a predominance of cutaneous leishmaniasis due to *Leishmania major* (*L. major*) (54%), with rodents as the reservoir and *Leishmania tropica* (*L. tropica*) (43%) with the man as a reservoir, and finally, visceral leishmaniasis due to *Leishmania infantum* (*L. infantum*) and the dog is its reservoir [3].

Geographically, the presence of CL in the different regions of the country is conditioned by the circulation of the *Leishmania* species in question. Therefore, the circulating species identified in the north is *L. infantum*; in the centre, it was *L. tropica*, while in the south the two species of *L. major* and *L. tropica* are responsible for the cases of cutaneous leishmaniasis [4]. Transmission is mainly anthroponotic in cases of *L. tropica* and zoonotic in cases of *L. infantum* and *L. major* [5–9].

Even though the fight against this dermatosis was officially launched in 1997 by the implementation of a national programme to control leishmaniases [10], the health authorities announced that the epidemiological situation of CL is still worrying and that their elimination must be maintained by 2030 to align with the health objectives of the 2030 sustainable development agenda in which Morocco was included [3].

At present, most of the work that was done on CL in Morocco focuses on the eco-epidemiological and parasitological aspects of the disease [7, 9, 11–13]. However, no studies were made to describe the sociocultural aspects of the disease. It is within this framework that the present study was conducted, for the first time notably in the centre of the country in the province of El Hajeb, to explore the beliefs and popular behaviours of individuals concerning cutaneous leishmaniasis and its vector.

The control and prevention of cases of CL attributed to *L. tropica*, whose disease reservoir is human, consists mainly of health education of the population at risk to protect themselves against the risk of sand fly bites and to consult in the event of the appearance of skin lesions in order to minimise and limit the circulation of the parasite between individuals. In central Morocco, where CL due to *L. tropica* circulates [10], the importance of awareness and education of people is essential to control and limit the spread of these dermatoses in these areas. Therefore, people suffering from CL should be encouraged to seek care from health professionals [14]. However, the perception of the disease and its treatment greatly influences how people use health services [14, 15]. The success of programmes for the prevention and control of any human disease depends on the active participation of the community. The involvement of the affected population is important to achieve the elimination goals of the leishmaniasis control programme. The programme must understand the popular beliefs and behaviours of individuals regarding CL and its vector as they are crucial determinants of community involvement. The results of this study can help health officials to improve the implementation of CL control activities in central Morocco. Moreover, the results obtained can be used to propose a regional plan adapted to the sociocultural context and appropriate to accelerate the process of CL elimination, especially in the central regions of the country.

The aim of this work is to explore beliefs and identify popular concepts surrounding the disease and its vector to understand the care-seeking behaviours of the population. The specific factors sought are knowledge of cutaneous leishmaniasis disease, attitudes and behaviours related to the treatment of skin lesions, mode of transmission, sand fly, and preventive measures. The objective of this study is to shed new light on the problem of cutaneous leishmaniasis by examining the beliefs and behaviours of individuals seeking care. Therefore, the results obtained can be applied to the prevention and control of CL insofar as they allow program decision-makers to initiate control activities adapted to the sociocultural context of the affected population in the central regions of Morocco and any other similar region.

## 2. Materials and Methods

### 2.1. Study Area

The study was conducted in the province (33°41'N, 5°22'W) located in central Morocco, at an altitude of 1000 m and a temperate climate. Annual rainfall varies between 400 mm and 600 mm [16]. It is bordered to the east by the province of Séfrou, to the west by the province of Khémissate, to the north by the prefecture of Meknes, and to the south by the province of Ifrane. It is a predominantly rural province with scattered groups of extended families attached to their farmland. With a population of 247,016 inhabitants and a surface area of 2193.41 km^2^, the province is organised into 16 communes, of which 12 are rural [17]. Economically and agroindustrially, the province of El Hajeb is celebrated nationally for its onion production (62% of national production), it is an important area for the central region of the country and offers employment opportunities throughout the year, and it is a meeting place for seasonal workers to participate in the cultivation and harvesting of onions (62% of national production) [18]. These workers come in particular from neighbouring provinces such as Fez, Séfrou, and Moulay Yacoub, which have recently declared as endemic foci of cutaneous leishmaniasis in central Morocco [13, 19, 20].

### 2.2. Epidemiological Data and Justification of the Study

Epidemiological data on human cases of leishmaniases were obtained from the health authorities at the delegation of the Ministry of Health at the level of El Hajeb province. Between 2013 and 2017, 21 new cases of leishmaniases were reported and were the subject of an epidemiological study [12]. The said study revealed the coexistence of the two forms of leishmaniasis with a predominance of the cutaneous form (81%) of the cases compared to 19% of the cases with visceral leishmaniasis. The first age of the disease infestation among the autochthonous cases was 13 months for LV and 24 months for CL. The majority of the reported cases are indigenous cases from rural areas (75%) of the new cases. 62% of these cases are notified in the communes of Bitit and Laqsir, which are located near the commune of Ain Chgag (province of Séfrou) [12]. The said study concluded that the displacement of people seeking seasonal employment, the increase of exchange with neighbouring provinces that are declared highly affected by CL, and the existence of ecological and environmental conditions favourable to the multiplication of disease-carrying insects and the proliferation of rodents increase the risk of the spread of CL disease in the population of El Hajeb, and education sessions will be necessary to prevent such a situation. But awareness about a disease first requires health professionals to have an idea about the population's knowledge and beliefs about the disease to correct misconceptions and improve true knowledge. Providing health actors with information on the level of knowledge of the population about the CL disease is therefore the ultimate goal of this study.

### 2.3. Study Design and Sampling

The study was based on the epidemiological data of leishmaniases in the province of El Hajeb to conduct an exploratory survey on the knowledge of CL by the population in the communes that were affected by this disease in recent years [12]. Therefore, the communes that were selected are Laqsir, Bitit, Ain Taoujdate, El Hajeb, Sabaa Ayoune, Bouderbala, Agourai, and Ait Oukhlifen. The survey was carried out between April and June 2019 door-to-door among 281 people in the city of El Hajeb.

The methodology followed for the design of the survey was that of Khan et al. [21], and the data collection tool was constructed following Frary's guidelines for a questionnaire [22]. The questionnaire consisted of four parts:

- The sociodemographic characteristics of the participants
- The population's knowledge of CL, its mode of transmission, and treatment attitudes
- Citizens' knowledge and beliefs about the sand fly and their ability to transmit diseases
- The measures used to avoid the disease

The information was collected using a pretested assisted questionnaire, translated into the local language, and validated with twenty-one people living in the city of Meknes and with sociodemographic characteristics similar to those of the population studied. The interviews were conducted door-to-door by nursing and health science students who were fluent in the city's two local dialects (Arabic and Berber). These students were familiar with the city and its inhabitants because of their frequent visits to the various health institutions during their work placements. Also, they are familiar with field research and how to conduct surveys scientifically and ethically, gathering the necessary information on the concepts used.

Participants were selected based on convenience sampling and their willingness to participate in the study. The criteria for inclusion were being adult, living in and originally from El Hajeb. While the exclusion criteria were refusal to participate in the survey, persons under 16 years of age, and those living in the city for less than six months.

Thus, the data obtained were entered into Excel, double-checked, and then transferred to Epi Info 7 for processing and statistical analysis. The results were presented as percentages and tables.

### 2.4. Ethical Considerations

Before conducting the study, we obtained the administrative authorization from the delegation of the Ministry of Health of the city of El Hajeb to carry out this survey. Also, all participants were informed about the objectives of the study and the full respect of the confidentiality of their identities and answers. They were, therefore, free to accept or refuse to participate in the study, as recommended by experts in scientific research ethics [23]. Thus, free and informed consent was obtained orally, and the interviews were carried out with only those people who freely agreed to participate in our study and to answer our questions, and after each interview, the participant received information about cutaneous leishmaniasis, its mode of transmission, and preventive measures.

## 3. Results and Discussion

Cutaneous leishmaniasis is a real public health problem, which has obliged the Ministry of Health to implement a national programme for the control of CL since 1997 [4]. Controlling the epidemiological situation of these diseases has become a national priority, particularly to achieve the health objectives of the 2030 sustainable development agenda in which Morocco is involved.

Despite efforts to control and eliminate CL at the national level, Morocco still suffers from the spread of these parasitoses. Most of the work that was carried out at the national level on CL focuses on the eco-epidemiological and parasitological aspects of the disease [6–8, 11–13]. To date, no research was carried out to study the sociocultural aspects of these parasitic dermatoses. It is within this framework that the results of this study are presented to describe, for the first time in Morocco, popular beliefs and behaviours of individuals concerning CL and its vector to better understand care-seeking behaviours.

### 3.1. The Sociodemographic Characteristics of the Participants

Table 1 presents the sociodemographic characteristics of the participants in terms of age, sex, marital status, education, occupation, and place of residence. Analysis of the results shows that women represent 68.0% and married persons represent 74.7% of the total number of participants. The age of participants ranges from 16 to 67 years, and the age group 41–50 has the highest participation rate of 26.7%. In terms of education level, the study sample revealed that 34.5% of participants have primary school education and 28.1% of participants are illiterate. In terms of the type of activity practised, housewives accounted for 27.0%, followed by those working in agriculture and livestock with a percentage of 20.8%.

Research results reveal that the rural population represents more than half of the participants (55.2%). Most of the respondents belong to poor families with a fragile social level and 55.9% have less than 3000 Moroccan dirhams per month, equivalent to US$300 per month. Also, 53.4% of the participants are vulnerable and have benefited from the Medical Assistance Regime for the Economically Diminished (MARED) and 22.8% have no medical coverage.

### 3.2. Behaviours of Citizens towards Cutaneous Leishmaniasis and the Use of Protective Measures

Table 2 presents the knowledge and behaviour of individuals regarding CL transmitted by sand fly. One of the most important results is that more than half of the participants have heard about CL (56.2%). But when pictures of skin lesions due to CL were shown, people were asked, “Have you ever seen a person with these skin lesions?” It turned out that the rate rose to 69.4%. Also, 50.8% said that these lesions are recognized by the popular name of “Hboub of Chniwla,” 12.8% said it is “Hboub of Namos,” 11.3% said it is “Hboub of Timsi/Bonif,” and 9.2% called it “Nar Lfarsiya,” and 3. 6% of them believe that these skin lesions are the result of witchcraft appearing on the skin of the victim “Tokal.” In the Moroccan dialect, the term “Hboub” refers to a lesion that affects the skin.

And on how a person is affected, 18.1% think the disease is caused by eating contaminated food. As for 24.6% of them, they do not know how the contamination occurs, while 34.9% say the infection was due to insect bites. This finding was confirmed by Koirala et al.'s study in Nepal, where they were able to show that most participants do not have a clear idea of how the disease is transmitted to humans [24]. Another study of CL knowledge and practice in southern Iran by Sarkari et al. showed that more than half of those affected by the disease believed that CL was caused by a microbe and not the *Leishmania* parasite [25]. In a very recent study conducted in the Volta region of Ghana, it was found that most of the inhabitants (88.3%) had satisfactory knowledge of CL but not of the vector and the mode of transmission [26].

Of the 98 people who confirmed that the CL lesions were caused by insect bites, 54.1% gave the name “Chniwla,” and 23.5% said it was “Namos,” i.e., mosquitoes, 7.1% said it was spiders, however, 5.1% said it was sand fly, and 2.5% did not know the name of the insect. This was consistent with the results of Sarkari and colleagues where they found that 63.5% of the inhabitants thought that mosquito bites could cause the disease and not sand fly [25]. In another study in Punjab, Pakistan, Akram and colleagues found that people often confused mosquitoes with sand fly as the true vectors of CL [27].

Regarding the severity of these skin lesions, the results showed that 33.1% of the participants did not consider them to be serious at all and 31.0% considered them to be relatively serious, while 25.3% did not know how serious they were. As for the curability of these lesions, 70.1% think that they could be cured, but more than a quarter (28.5%) did not know if they were curable or not. It was interesting to note that to treat CL, the population used traditional therapeutics (20.6%), applied basil and vinegar-based recipes instead of the bite (23.1%), and used substances on skin lesions such as bleach (sodium hypochlorite) and perfumes, while 44.1% reported that they were treated by health care professionals. This finding was consistent with similar studies conducted in Suriname by Ramdas who found that patients with skin lesions used hazardous chemicals to treat CL [28]. In another recent study conducted by Doe in Ghana, it was found that most people believed that CL is primarily cured by a local type of herb [26].

From the Moroccan sociocultural point of view, these results could be explained, on the one hand, by the fact that the practices of the inhabitants of the city of El Hajeb in the centre of the country for the treatment of CL lesions did not differ from the results found by Bennis and his colleagues in the provinces of “Tinghir” and “Errachidia” in the south of Morocco, who also used traditional treatments and self-medication practices [29]. On the other hand, these behaviours revealed gaps and inadequacies in the knowledge of Moroccans and their misconceptions about the effectiveness of traditional treatments despite the free and available medical treatment in health centres. This revealed the importance of implementing health awareness and education strategies.

Concerning the possibility of prevention against CL, the results obtained showed that 31.5% of the participants think that CL was not preventable and 6.8% did not know if it is possible to prevent this disease. On the other hand, 61.9% stressed the possibility of prevention. In this context, when we asked the question “How can prevention be done and what was the method?”, it turned out that 29.9% thought that prevention consists in avoiding insect bites, 22.1% thought that prevention consists in avoiding contact with people with this disease, and 4.3% said that prevention consists in isolating the sick person. These findings corroborated with Abazid's study in Aleppo, Syria, who found that most residents used mosquito nets to protect themselves from bites and did not believe that the disease is contagious [30].

Cutaneous leishmaniasis, as a lesion affecting the skin, can leave permanent disfigurement and negative impacts on the psychological and social life of the affected people [29]. The benign nature of dermatosis often classifies it as a secondary rodent compared to other fatal diseases. As a result, the CL is considered by the WHO as a tropical disease neglected by health systems [31]. Nevertheless, this neglect can also be seen in individuals themselves. When we asked the question: “Do you think that skin lesions require the consultation of a health professional?” (Table 3), the results show that 32.4% of the participants answered “no” and 5.0% did not know if these skin diseases required medical consultation, while 55.5% of respondents indicated that skin diseases require the advice of a health professional.

And when we asked them: “Can skin diseases be a health problem?”, it turned out that a third of the participants (32.7%) did not consider them to be a health problem, while 65.7% responded that they could be a problem if the skin lesion was in a hidden area of the body such as the genitals (94.0%), or when it was in an exposed area of the body such as the face (93.5%). We also found that skin diseases are related to gender, as the majority of respondents reported that they can be a health problem for women. Similarly, skin diseases are related to childhood, as they are considered a health problem if they affect young children, regardless of gender: 97.3% for boys and 98.9% for girls. These results can be explained by the fact that the perceptions of the inhabitants of central Morocco about these skin lesions are the same as those of the inhabitants of the province of San José in Costa Rica because they only consider CL as a health problem if their children are affected [32]. Also, CL is not considered a health priority for the population in the south of the country compared to the many competing health problems [29].

### 3.3. Citizens' Knowledge and Beliefs about Sand Fly

Table 4 presents the results of research on citizens' knowledge and beliefs about a sand fly, where they live and reproduce, and their ability to transmit diseases. Among the important results obtained, the majority of participants know sand fly under the name “Chniwla” (61.6%) and the name “Namos” (34.5%). And when we asked them: “Is there a difference between sand fly and mosquitoes?”, it turned out that 69.8% of the participants thought there was no difference and 3.2% said they did not know. These results confirm those reached by Bennis and his colleagues when they carried out their qualitative research on the psychological effects of CL in south-eastern Morocco in the regions of Tinghir and Errachidia, where it was discovered that the victims of CL use the names “Chniwla” and “Namos” to designate the insect that bit them and caused the skin scars [29]. Therefore, it can be said that the local name of sand fly in Morocco does not differentiate between its interior and southern regions [14]. Also, the lack of distinction between mosquitoes and sand fly was not only observed among Moroccans but was also raised among residents of Punjab, Pakistan, where participants were unable to distinguish between these two small insects [27]. In Sri Lanka and Ecuador, the degree of mixing was important because no distinction was made between sand fly and mosquitoes and they were used as synonyms [24, 33].

About the knowledge of the place of reproduction and proliferation of sand fly, several studies revealed the erroneous knowledge of individuals [34, 35]. This was confirmed in our resultants since 41.3% of the participants believe that sand fly develops in water, especially polluted water (22.8%) or waste and garbage disposal places (45.6%). But only 17.1% said that the manure of the animals is the place of reproduction of these insects and 16.7% of the respondents did not know where they reproduce.

Knowledge of individuals at risk of disease transmission by sand fly is an essential factor in ensuring primary prevention of CL. Our results show that 44.1% of respondents believe that sand fly is incapable of transmitting diseases, 22.1% do not know if it is capable of doing so, while only 33.8% of respondents indicated that these insects can transmit diseases. And for those who have already recognized the pathogenic capacity of the sand fly, 80.0% of them say that it transmits diseases to humans and animals, while 4.2% think that it transmits diseases to trees and plants.

According to participants, sand fly-borne diseases include skin diseases (91.6%), allergic diseases (37.9%), fever (24.2%), skin leishmaniasis (22.1%), and eye diseases (17.9%). In a similar study conducted in Pakistan by Akaram and colleagues with 250 people, they concluded that only a few participants (9.2%) thought that sand fly transmitted CL and the others thought that it transmitted fever, diarrhoea, respiratory, and skin diseases [27].

Concerning information and awareness of people about the health risks of the sand fly, we asked our participants: “Have you ever been informed about diseases transmitted by a sand fly?” And if the answer is yes, then who has been? It turned out that 64.8% had never been informed before. Among those who answered yes, the Internet was their means of obtaining information (47.5%), followed by neighbours and friends (45.5%). This finding was also found in Fernando's study in Colombia on knowledge about CL and its vector, where he found that most people were informed about the disease through their family and colleagues [33].

### 3.4. Preventive and Protective Measures against the Risks of Sand Fly

Table 5 shows the methods most commonly used by citizens to protect themselves against the risks associated with sand fly infestations. We asked our participants the following question: “In your opinion, can sand fly be avoided?” It turned out that more than half (52.7%) thought that these insects could not be avoided, while 42.7% said that sand fly bites could be avoided by using basil plants (90.8%) and mint (15.8%). However, others indicated the effectiveness of powdered pesticides (55.8%), vinegar (60%), and lemon (45.8%) and only 20% of participants indicated respect for environmental health as a means of prevention. However, the scientific methods that are recommended to protect against sand fly bites are completely different from those mentioned by respondents. This is why we have proposed to choose one of the methods that should be used in the prevention, namely, mosquito nets, long clothing, and insecticides. The results obtained reveal that more than half of the participants prefer to use an insecticide in the form of the insecticide pump (69.8%) and mosquito nets (46.6%). Although the use of long-lasting pesticide-treated nets was recommended in the fight against cutaneous leishmaniasis by WHO [1], their use was only mentioned by 8.9%.

Thus, the analysis of the results on the role of individuals in the fight against disease-carrying insects shows that 26% thought that it was the responsibility of the Ministry of Health, 18.5% thought that it was the role of the Ministry of Agriculture, while only 6.4% said that it was the individual himself or herself who was responsible for vector control. Indeed, WHO recommendations indicate that sustainable control of the spread of vector-borne diseases requires active community participation and a holistic approach that ensures the integration of all sectors including the Ministry of Health, Ministry of Agriculture, Ministry of Education, and individuals. WHO emphasizes the importance of tailoring prevention and control measures to the context of each region and using simple, cost-effective procedures that improve the quality of people's lives, such as the use of nets, health education, early diagnosis, and immediate treatment [1, 31].

## 4. Conclusions

By way of conclusion, the present study has revealed, on the one hand, the gaps in knowledge and the misconceptions of individuals about the disease, the vector, the mode of transmission, and the prevention measures. On the other hand, to reveal the negligent behaviours towards leishmaniasis skin lesions that surround the care-seeking practices for this type of dermatosis. This underlines the importance of implementing health education campaigns to increase awareness of cutaneous leishmaniasis. Health awareness and increasing the level of people's awareness of cutaneous leishmaniasis and its vector can be improved by using the popular concepts obtained to simplify scientific terms and adapt them to the culture of the population. This facilitates the understanding of educational messages on the prevention of cutaneous leishmaniasis by individuals and actively integrates them in the process of controlling leishmaniasis in central Morocco and regions with a similar situation.

## Figures and Tables

**Table 1:** Sociodemographic characteristics of participants.

	Variable	Number (*n* = 281)	Percentage (%)
Middle of life	Urban	126	44.8
	Rural	155	55.2

Age group	[16–20]	17	6.0
	[21–30]	61	21.7
	[31–40]	69	24.6
	[41–50]	75	26.7
	Over 50 years old	54	19.2
	Unanswered	5	1.8

Level of education	Illiterate	79	28.1
	Primary	97	34.5
	College	62	22.1
	Secondary	23	8.2
	Superior	17	6.0
	Unanswered	3	1.1

Occupation	Housewife	76	27.0
	Public servant	33	11.7
	Farmer/breeders	58	20.8
	Worker	28	10.0
	Works day labourer	45	16.0
	Other occupations	41	14.6

Gender	Man	91	32.0
	Woman	191	68.0

Family status	Unmarried	46	16.4
	Married	210	74.7
	Widower	16	5.7
	Divorced	9	3.2

Monthly family income	<300$	157	55.9
	[300–500]	69	24.6
	[500–700]	38	13.5
	>700$	17	6.0

Health coverage	National fund for social welfare	23	8.2
	National social security fund	44	15.7
	Medical assistance regime for the economically disadvantaged	150	53.4
	None	64	22.8

Housing type	A traditional house made of wood, brick, and pewter	64	22.8
	A traditional cement house	121	43.1
	Modern Moroccan house	71	25.3
	Apartment	23	8.2
	Villa	2	0.7

**Table 2:** Citizens' knowledge and beliefs about cutaneous leishmaniasis.

Question	Answer	Number (*n* = 281)	Percentage (%)
Have you heard of cutaneous leishmaniasis?	Yes	158	56.2
	No	123	43.8

Have you ever had or seen anyone with such skin lesions? (picture of cutaneous leishmaniasis)	Yes	195	69.4
	No	82	29.2
	No answer	4	1.4

What do you call these skin lesions?	Hboub Chniwla	99	50.8
	Hboub Namos	25	12.8
	Hboub timssi/Hboub Bonif	22	11.3
	Nar Lfarsiya	18	9.2
	Sorcery (magic)	7	3.6

In your opinion, are these skin lesions considered?	Very serious	11	3.3
	Serious	19	6.8
	Mildly severe	87	31.0
	Never mind	93	33.1
	I do not know	71	25.3

In your opinion, are these skin lesions curable?	Yes	197	70.1
	No	4	1.4
	I do not know	80	28.5

If yes, how is it possible? (you can answer several choices)	Through treatment by a health care professional	124	44.1
	By treatment at the pharmacist's	34	12.1
	Use of home-prepared self-treatment recipes	65	23.1
	By remedies from the traditional healer	58	20.6

In your avis, how can cutaneous leishmaniasis be transmitted to humans? (you can answer several choices)	By insect stings	98	34.9
	By consuming contaminated food	51	18.1
	By the polluted water	26	9.3
	By the polluted air	12	4.3
	By an animal bite	9	3.2
	Other means	16	5.7
	I do not know	69	24.6

If cutaneous leishmaniasis is transmitted to humans by insects, what kind of insects?	Chniwla	53	54.1
	Namos (mosquitoes)	23	23.5
	Spiders	7	7.1
	Sand fly	5	5.1
	Other insects	8	8.1
	I do not know	2	2.0

In your opinion, is this skin disease preventable?	Yes	174	61.9
	No	88	31.3
	I do not know	19	6.8

If yes, what is the method? (you can answer several choices)	Avoid approaching or touching the person with the disease	62	22.1
	Avoid infection by isolating the affected person	12	4.3
	Avoid sick animals	16	5.7
	Avoid insect stings	84	29.9

**Table 3:** Citizens' views on skin lesions.

Question	Answer	Number (*n* = 281)	Percentage (%)
In your opinion, do dermal diseases require the advice of a health professional?	Yes	156	55.5
	No	111	39.5
	I do not know	14	5.0

In your opinion, can dermal diseases be a health problem for the individual?	Yes	184	65.7
	No	92	32.7
	I do not know	5	1.8

If so, for whom could skin disease be a health problem? And in what area of the body? (you can answer with several choices)	For the man	63	34.2
	For the married woman	76	41.3
	For the unmarried woman	85	46.2
	For the little girl	182	98.9
	For the little boy	179	97.3
	If the skin lesion is in a visible area of the body	175	95.1
	Feet	51	27.7
	Hands	42	22.8
	The face	172	93.5
	Another skin lesion is in a visible area^*∗*^	27	14.7
	If the skin lesion is in a hidden area of the body	178	96.7
	The abdomen	75	40.8
	The back	50	27.2
	The genitals	173	94.0
	Another skin lesion is in a hidden area^∗∗^	92	50.0

^*∗*^The scalp; ^∗∗^breasts.

**Table 4:** Citizens' knowledge and beliefs about the sand fly.

Question	Answer	Number (*n* = 281)	Percentage (%)
In your opinion, is there a difference between sand fly and mosquitoes?	Yes	196	69.8
	No	76	27.0
	I do not know	9	3.2

What do you call sand fly?	Chniwla	173	61.6
	Namos	97	34.5
	I do not know	11	3.2

Do you know the breeding grounds of sand fly?	Water of all kinds	116	41.3
	Stagnant water	32	11.4
	Polluted water	64	22.8
	Damp places	19	6.8
	Dry places	3	1.1
	Cold places	22	7.8
	Dirty and polluted places	81	28.8
	Waste and garbage disposal places	128	45.6
	Human excrement	62	22.1
	Animal manure	48	17.1
	I do not know	47	16.7

Have you ever received information on diseases transmitted by sand fly?	Yes	99	35.2
	No	182	64.8

If yes, from whom?	Internet	47	47.5
	Friend/Neighbour	45	45.5
	Television	39	39.4
	Health care professional	24	24.2
	Radio	6	6.1
	School	2	2.0

In your opinion, can sand fly transmit diseases?	Yes	95	33.8
	No	124	44.1
	I do not know	62	22.1

If yes, to whom can diseases be transmitted?	The human	67	70.5
	The animal	26	27.4
	Human and animal	76	80.0
	Trees and plants	4	4.2
	I do not know	19	20.0

What are these diseases?	Skin diseases	87	91.6
	Allergy	36	37.9
	Fever	23	24.2
	Cutaneous leishmaniasis	21	22.1
	Eye diseases	17	17.9
	Diarrhoea	7	7.4
	Malaria	3	3.2
	Asthma	2	2.1
	Influenza	2	2.1
	Yes, but I do not know the name of the disease	7	8.4

**Table 5:** Measures used by citizens to protect themselves against insect bites.

Question	Answer	Number (*n* = 281)	Percentage (%)
In your opinion, the control of disease-carrying insects is a responsibility of (you can answer with several choices)	The Ministry of Health	73	26.0
	The Ministry of Agriculture	52	18.5
	Ministry of the Interior (municipality)	30	10.7
	The three departments	24	8.4
	The person himself	18	6.4
	Associations and NGOs	10	3.6
	Shared responsibility among stakeholders	60	21.4
	I do not know	8	2.8

In your opinion, can sand fly be avoided?	Yes	120	42.7
	No	148	52.7
	I do not know	13	4.6

If yes, how is that possible? (you can answer with several choices)	Basil	109	90.8
	Vinegar	72	60.0
	Pesticide (powder)	67	55.8
	Lemon	55	45.8
	Mint	19	15.8
	Cleanliness	24	20.0

To protect yourself from insect bites, use yourself (you can answer with several choices)	Pesticide pump	196	69.8
	Plants	147	52.3
	Curtains in the windows	131	46.6
	Wear long-sleeved shirts	70	24.9
	Curtains at the doors	65	23.1
	Wearing pants	57	20.3
	Electrical distributors for pesticides	50	17.8
	Screens in doors and windows	48	17.1
	Fan (air conditioner)	36	12.8
	Insecticide-impregnated mosquito nets	25	8.9
	Mosquito nets around the bed	5	1.8
	Other means	38	13.5

## Data Availability

The data used in this study are included within the article.
